# Supplementary material for: Alpha-Synuclein FRET Biosensors Reveal Early Alpha-Synuclein Aggregation in the Endoplasmic Reticulum
Source: Life (Basel). 2020 Aug 11;10(8):147. doi: 10.3390/life10080147 (PMC7460339; doi:10.3390/life10080147)
Supplement: Supplementary file 1 [file life-10-00147-s001.pdf]

life

MDPI

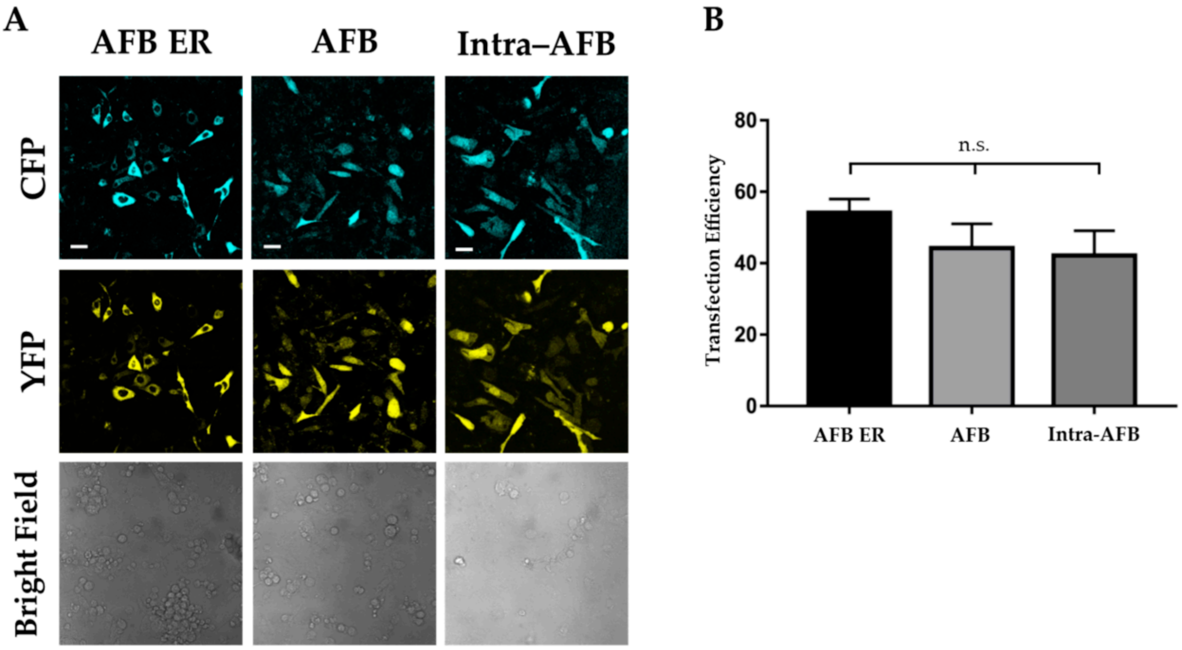

Figure S1. Transfection efficiency of AFBs in i36 cells in untreated conditions. AFBs were expressed in i36 cells and transfection efficiency was assayed as ratio of CFP or YFP positive cells over total number of cells in the bright field. (**A**) Confocal images of i36 cells transfected with Inter-AFB ER, Inter-AFB or Intra-AFB were acquired in bright field, in CFP and YFP channel with a Leica confocal microscope SP2, using a 40 x oil objective. Scale bar, 10 µm. (**B**) Data analysis shows similar transfection efficiency for all AFBs. Graph values represent percentage of transfection efficiency and are expressed as mean ± SEM (n = 3 or 4). n.s., not significant.
